# Cytokines, Chemokines, and Chemokine Receptors Quantitative Expressions in Patients with Ovarian Cancer

**Published:** 2015-05

**Authors:** Somayeh Rezaeifard, Mahboobeh Razmkhah, Minoo Robati, Mozhdeh Momtahan, Abbas Ghaderi

**Affiliations:** 1 Shiraz Institute for Cancer Research, School of Medicine, Shiraz University of Medical Sciences, Shiraz, Iran;; 2 Department of Immunology, School of Medicine, Shiraz University of Medical Sciences, Shiraz, Iran;; 3 Department of Gynecology, School of Medicine, Shiraz University of Medical Sciences, Shiraz, Iran

**Keywords:** Ovarian cancer, Ovarian cyst, Chemokines, Cytokines

## Abstract

**Background:**

Cytokines, chemokines, and chemokine receptors regulate the proliferation and survival of tumor cells, angiogenesis, and metastasis to other organs. This network of ligands and receptors has been used in molecular targeting of cancer.

**Methods:**

We compared the mRNA expression of CXCR3, CXCL-10, CXCR4, CXCL-12, IL-4, and IL-10 in tissues of benign and malignant ovarian tumors by qRT-PCR method and evaluated serum IL-10 and CA-125 content of these patients by ELISA during one year.

**Results:**

Our result showed a trend toward a higher expression of CXCR4 in malignant ovarian tissues compared with the benign ovarian cysts (P>0.05). However, SDF-1, IP-10, IL-4, CXCR3, and IL-10 had a lower trend in mRNA expression in malignant ovarian tissues compared to the benign cyst tissues. Except for IL-4 (P=0.01) and SDF-1 (P=0.02), the data for other factors were not statistically significant. A trend toward higher concentration of IL-10 was observed in the serum of ovarian cancer patients compared to those with benign cysts; however, the difference was not significant. CA-125 concentration in the serum of ovarian cancer patients was higher than that of benign cyst patients (P=0.05).

**Conclusion:**

According to results obtained, we hypothesize that the lower expression of SDF-1 in malignant tissues may have an important role in ovarian tumor growth. However, this hypothesis requires more investigation. Higher levels of CA125 and IL-10 in the serum of patients might indicate that the combination of these biomarkers could be used for distinguishing patients with ovarian cancer from those with benign cysts.

## Introduction

The fifth cause of death from malignancies in women with a high rate of mortality is ovarian cancer.^1^ Over the past two decades, several studies have examined the roles of cytokines and chemokines in the promotion of many tumor types. For example, tumor cells inhibit antitumor immune response by secreting inhibitory cytokines like Interleukine-10 (IL-10).^2^ High IL-10 mRNA levels promote cell growth and migration in the malignant oral squamous cell carcinomas.^3^ The immunosuppressor effects mediated by this cytokine may support an environment favorable to neoplastic cell expansion.^4^ High levels of IL-10 are found in the serum and ascites of ovarian cancer patients.^5^ IL-10 levels consistently correlate with advanced disease and poor patient prognosis in ovarian cancer^6^^,^^7^ and other types of cancer including lymphoma^8^ and lung cancer.^9^ IL-10, Interferon-gamma-inducible protein-10 (IP-10), Monocyte chemoattractant protein-1 (MCP-1) and Stromal cell-derived factor-1 (SDF-1) were significantly higher in B-cell lymphoma patients than in control subjects, suggesting the involvement of these mediators in the pathophysiology of B-cell lymphoma.^10^ Another study has demonstrated that high expression of CXCR3 can promote metastasis of melanoma cells to the lymph nodes.^11^ Over expression of CXCR4 is known in many tumor types such as prostate^12^ and renal cell carcinoma.^13^ SDF-1 expression has been also indicated to be high in breast^14^ and colorectal cancer, leading to shorter survival.^15^ By contrast, upregulation of SDF-1 in tumors may reduce tumor growth by attracting dendritic cells to tumor sites.^16^ Similar to other tumor types, chemokines and their receptors regulate the proliferation and survival of ovarian tumor cells, angiogenesis, and metastasis to other organs. Recently, the production of some cytokines in the ovarian cancer microenvironment has been explained as an autocrine cytokine network. This network includes tumor necrosis factor alpha (TNF-α), Interleukine-6 (IL-6) and CXCL12 (17). Notably, the CXCL12/CXCR4 axis play a major role in the promotion of ovarian cancer.^18^ SDF-1 production by malignant epithelial cells has a role in tumor genesis in the epithelial ovarian cancer.^19^

CA-125 (cancer antigen-125) is a glycoprotein that is ubiquitous on the surface of the normal mesothelial cells lining the body cavities.^20^ High cell surface expression of CA-125 is reported in tumors such as ovarian cancer, mesothelioma and some other cancers.^21^ This molecule is also released into the circulation and its serum level is used for assessing disease progression in ovarian cancer. Additionally, it is elevated in mesothelioma and some benign conditions.^22^ In spite of the high sensitivity of CA-125 in ovarian cancer, biased increased levels of this marker are illustrated in benign conditions. In the current study, we performed a comparative analysis on mRNA expression of CXCR3, CXCL-10 (IP-10), CXCR4, CXCL-12 (SDF-1α), IL-4 (interleukine-4) and IL-10 in tissues of benign and malignant ovarian tumors by the qRT-PCR method. In addition, IL-10 and CA-125 contents were measured in the serum of those patients to evaluate if the combination of these molecules would be able to differentiate ovarian cancer from ovarian cyst. The profile of these cytokines/receptors as well as simultaneous measurement of IL-10/CA-125 has not been investigated in Iranian patients with ovarian cancer and not compared to those with benign cysts.

## Materials and Methods

*Patients*

Serum samples were obtained from 47 patients from southern Iran, among whom 27 suffered from malignant ovarian cancer and 20 were diagnosed with benign cysts. Additionally, malignant and benign cyst tissues of ovaries were obtained from 12 and 16 patients, respectively for RNA extraction and quantitative real time polymerase chain reaction (qRT-PCR). The sample size was determined by a statistician using the following formula: 

n=(t^ 2^ ×p(1-p))/m^ 2 ^, where t=1.96 (95% confidence interval), m=0.05 and p=allele frequencies.

Samples were collected from Shahid Faghihi Hospital, Shiraz University of Medical Sciences (Shiraz, Iran), during a one-year period. The mean and median ages of ovarian cancer patients were 39.8±19.6 and 42.5, respectively. Patients were not undergoing any clinical treatment such as chemotherapy at the time of tissue specimen resection. Five patients were diagnosed with pathological stage I, two with pathological stage II, eighteen patients with pathological stage III and two patients with pathological stage IV malignant ovarian cancer. The data of ovarian cancer patients were compared to those of benign ovarian cyst patients in a cross-sectional study. The mean and median ages of individuals with benign cysts were 43.5±19.9 and 43, respectively. This study was approved by the Ethical Committee of Shiraz University of Medical Sciences and all samples were obtained after giving consent.

*RNA Extraction, cDNA Synthesis and Reverse Transcription*

Total RNA was extracted from tissues by Invisorb RNA kit II (Germany). cDNA was synthesized from the extracted RNAs by the cDNA synthesis kit based on the manufacturer’s instructions (Fermentas, Lithuania). The quantities of CXCR3, IP-10, CXCR4, SDF-1α, and IL-4 and IL-10 gene transcripts were determined by a Bio-Rad thermal cycler (Chromo4 Real-time PCR Detector, Bio-Rad, USA) for quantitative real-time PCR (qRT-PCR). Each PCR reaction was done in a final volume of 20 μl that contained 2 µl cDNA, 10 µl of 2X SYBR Green Master Mix (Fermentas, Lithuania), 0.3 µl of each 10 pmol forward and reverse primers (table 1) and 7.4 µl DEPC treated water. PCR amplification was carried out in 50 cycles using the following program: 95°C for 10 minutes, 95°C for 15 seconds, 56°C for 20 seconds and 60°C for 1 minute. All data were compared with those from beta actin housekeeping gene. Expressions of CXCR3, IP-10, CXCR4, SDF-1α, IL-4, and IL-10 mRNAs in tissues obtained from patients were determined using 2^-∆CT^ method.

**Table 1:** Primers used for qRT-PCR

**Gene**	**Primer sequence**
Beta actin	Reverse: CACCATCACGCCCTGGTGCC
	Forward: ACAGAGCCTCGCCTTTGCCG
CXCR3	Reverse: CAGTCACTGCTGAGCTGGAG
	Forward: AGCTCTGAGGACTGCACCAT
CXCR4	Reverse: TGGAGTGTGACAGCTTGGAG
	Forward: GGTGGTCTATGTTGGCGTCT
IL-4	Reverse: AGCAGTTCCACAGGCACAAG
	Forward: AGCAGTTCCACAGGCACAAG
IL-10	Reverse: CTGGAGTACAGGGGCAGTAT
	Forward: TGGTGAAACCCCGTCTCTAC
IP-10	Reverse: CAAAATTGGCTTGCAGGAAT
	Forward: AGGAACCTCCAGTCTCAGCA
SDF-1α	Reverse: CAGCCGGGCTACAATCTGAA
	Forward: TGCCAGAGCCAACGTCAAG

*Enzyme Linked Immunosorbent Assay (ELISA)*

IL-10 and CA-125 concentrations were evaluated using ELISA. Human IL-10 ELISA Kit from Bender MedSystems was used to measure IL-10 concentration in the serum of ovarian cancer and benign cyst patients. Briefly, the samples were added to the wells. Biotinylated anti-IL-10 was added to each well; the plates were incubated at room temperature (RT) for 2 hours, and then washed 3 times. Strepavidin conjugated enzyme was added to each well. After 1 hour of incubation at RT, 100 µl TMB substrate solution was added to each well. Enzymatic reaction was stopped with 100 µl of stop solution and the absorbance was measured at 450 nm by ELISA reader. 

Human CanAg CA-125 EIA Kit from Fujirebio Diagnostics EIA Kits was used to measure the CA-125 content in the serum of ovarian cancer and benign cyst patients. Briefly, the samples were added to the wells. Biotinylated anti-CA-125 was added to each well, and the plates were incubated at RT for 2 hours and then washed 3 times. Tracer working solution was added to each well. After 1 hour of incubation at RT and washing 6 times, 100 µl TMB-HRP substrate solution was added to each well. Then it was incubated for 30 minutes at RT and immediately the absorbance was measured at 620 nm in a microplate spectrophotometer. 

*Statistical Analysis*

Statistical analysis was performed using SPSS software (version 11.5; SPSS Inc., Chicago, IL, USA). Analysis of gene expression as well as IL-10 and CA-125 serum concentrations between the malignant and benign patients were considered significant if P values were ≤0.05 using Mann–Whitney nonparametric test. Graphs were prepared using GraphPad Prism 5. 

## Results

**Figure 1:**
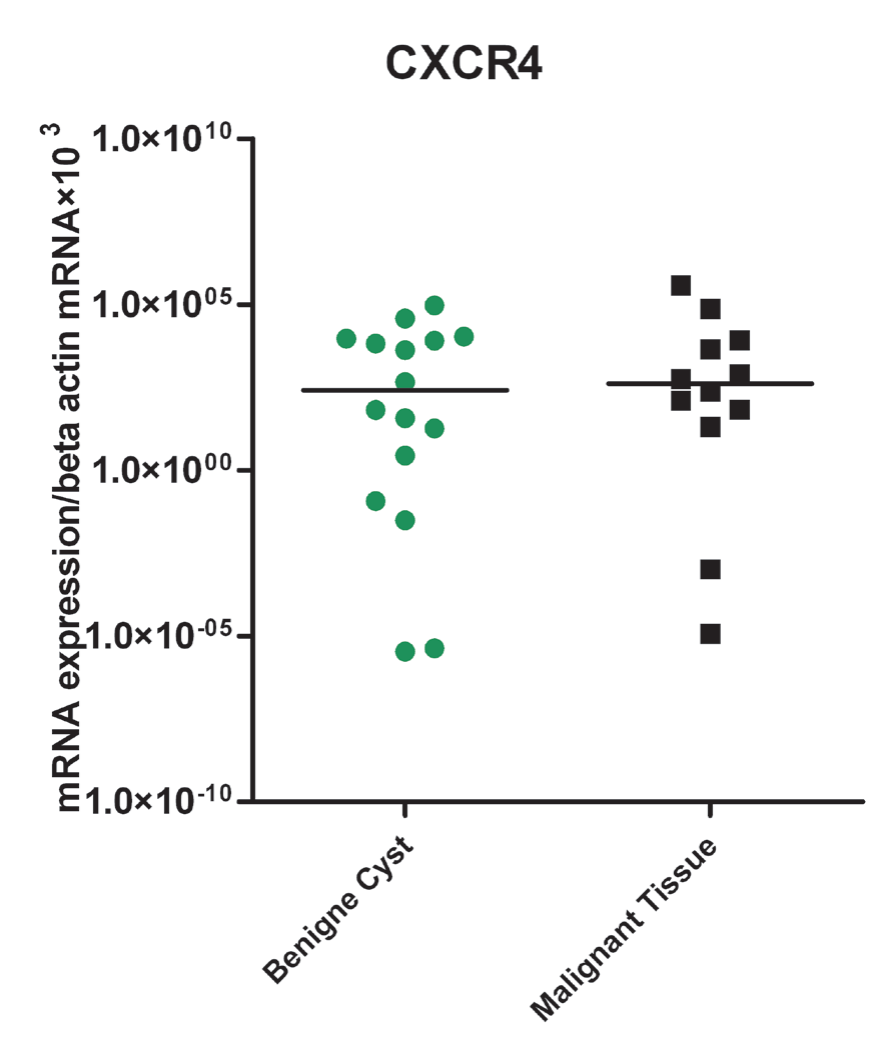
Expression of CXCR4 in tissues of benign cysts and malignant ovarian cancer patients.

The results showed a trend toward higher expression of CXCR4 in malignant tissues compared to ovarian benign cysts (Median=408.75 vs. 261.49 respectively), however, this difference was not statistically significant (P=0.6, figure 1).

**Figure 2:**
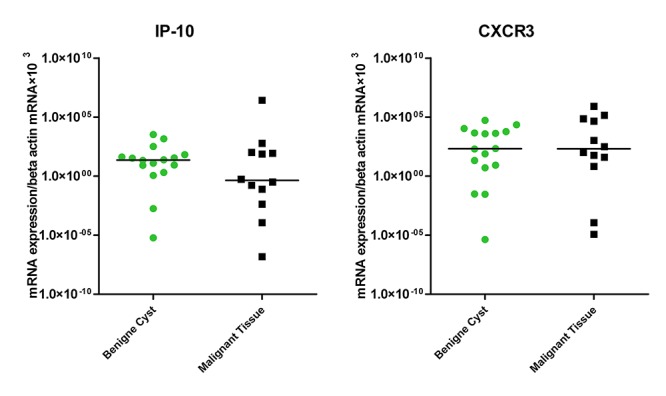
Expression of IP-10 and its receptor in tissues of benign cysts and malignant ovarian cancer patients.

In spite of a trend toward higher CXCR4 expression in malignant tissues, its ligand, SDF-1α, had a much higher expression in benign cysts (Median=9.4625 vs. 807.313 respectively). The difference was statistically significant (P=0.02; figure 2).

**Figure 3:**
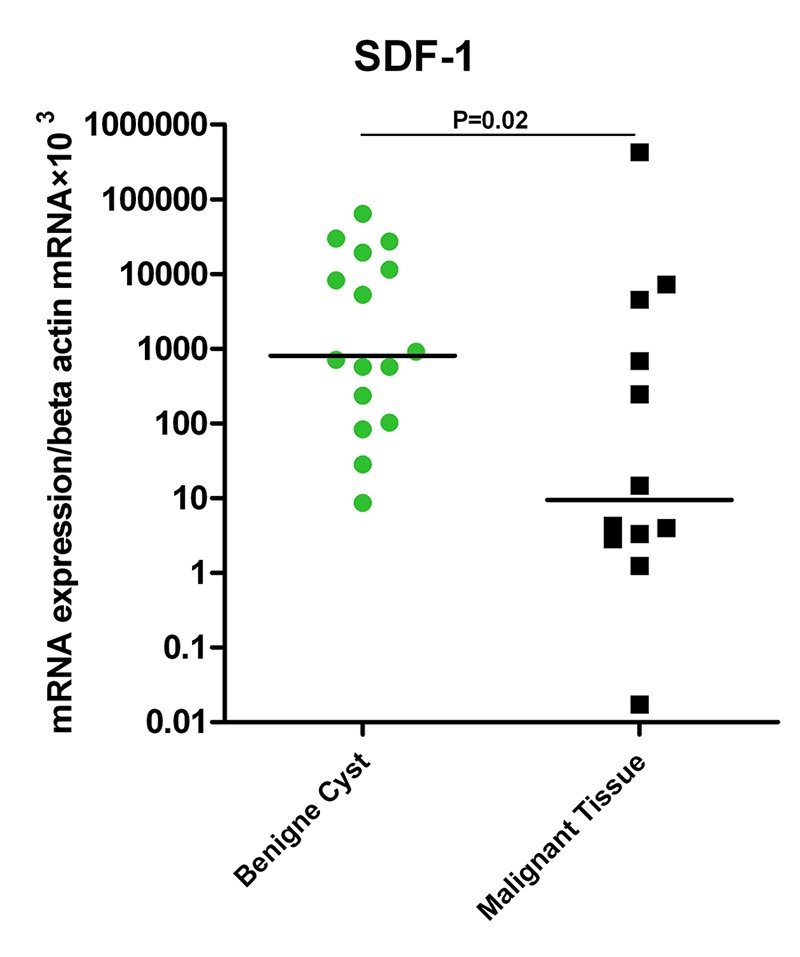
Expression of SDF-1α in tissues of benign cysts and malignant ovarian cancer patients.

Another chemokine/chemokine-receptor, CXCR3/IP-10, did not show any significant difference in expression (P>0.05) both in benign and malignant tissues (figure 3).

**Figure 4:**
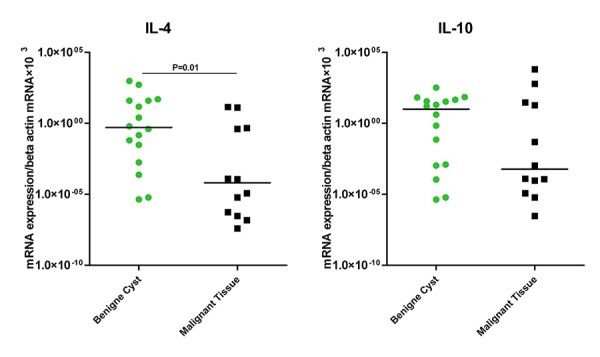
Expressions of IL-4 and IL-10 in tissues of benign cysts and malignant ovarian cancer patients.

In addition, IL-4 with a median of 0.00006 in malignant patients had a statistically lower mRNA expression compared to a value of 0.5 for benign cyst subjects (P=0.01). IL-10 (with a median mRNA expression of 0.0005 for malignant cancer patients vs. 9.971 for benign cyst subjects) demonstrated no significant difference (figure 4).

**Figure 5:**
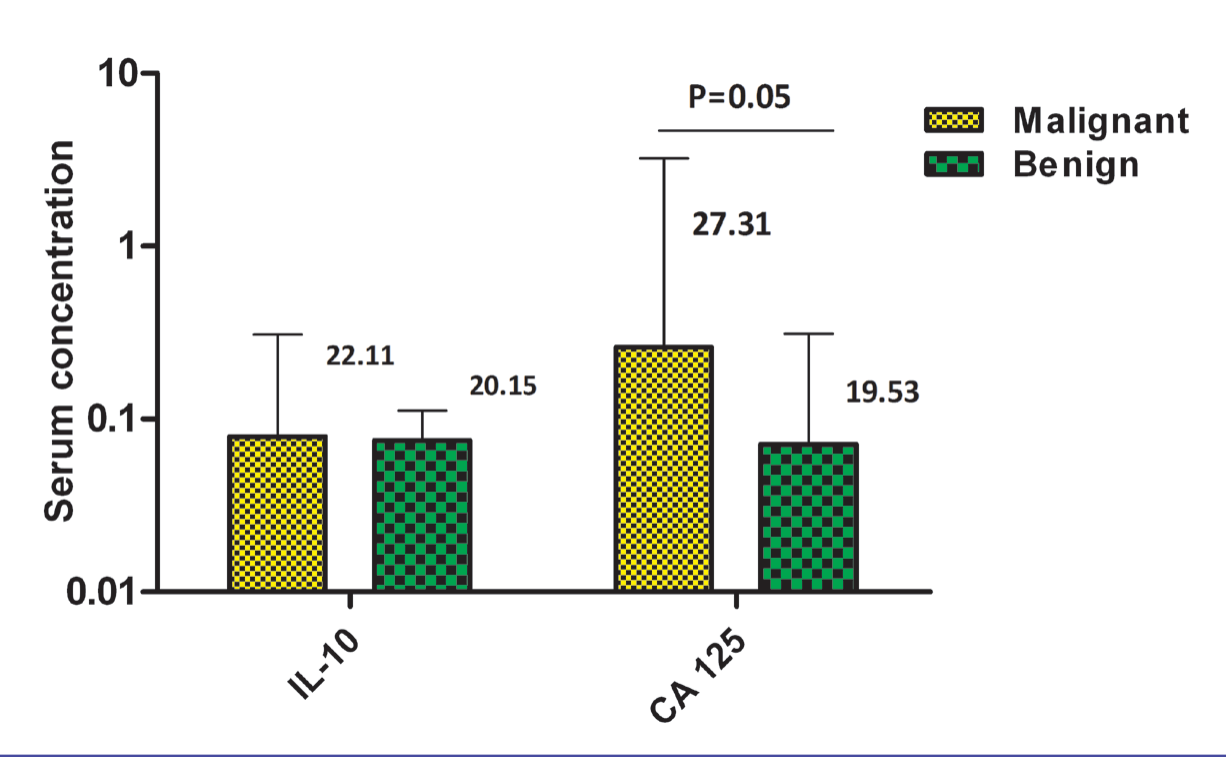
Comparison of the serum concentrations of IL-10 and CA-125 in ovarian cancer and benign cyst patients.

The serum concentrations of IL-10 and CA-125 are presented in figure 5. A trend toward a higher concentration of IL-10 was observed in serum of ovarian cancer patients compared to those with benign cysts. However, this difference was not statistically significant (P=0.08). CA-125 concentration in the serum of ovarian cancer patients was higher than that of benign cyst patients. This difference was statistically significant (P=0.05).

## Discussion

Survival in ovarian cancer patients remains poor as they usually are diagnosed during high stages. Also, most of the patients demonstrate recurrence of cancer after chemotherapy.^23^ Therefore, new targets and strategies should be identified to improve the results of treatment in ovarian cancer patients. There are growing evidence that chemokine CXCL12 (stromal-derived factor-1) and its receptor CXCR4 mediate invasiveness and metastasis of many types of cancer cells, such as prostate, bladder, breast and pancreas.^24^ Also, Many studies have shown that this chemokine and its receptor may have an important role in the progression of ovarian cancer.^18^ As mentioned above, we found a trend toward higher expression of CXCR4 in malignant ovarian tissues compared to the benign cysts. However, mRNAs of SDF-1α, IP-10, IL-4, CXCR3, and IL-10 had a trend toward lower expression in malignant ovarian tissues, among which expressions of IL-4 and SDF-1α were statistically significant. These results probably provide more evidence for a connection between CXCR4 expression and progression of ovarian cancer. A weakness of this study is the low number of cases, which is a reflection of the fact that ovarian cancer is not very common in our region. Therefore, to obtain a consolidated data, investigation is required to be carried out on a larger sample size. Signaling from CXCR4 can promote angiogenesis,^25^ cancer cell proliferation and metastasis to other organs.^26^ Anti-CXCR4 blockade antibody attenuated breast cancer cell migration^27^ and also decreased non-small cell lung cancer metastasis.^28^ Therefore, CXCR4 can be a potential target for the immunotherapy of ovarian cancer. CXCR4 blocking antibody may inhibit the signaling pathways, metastasis and tumor cell proliferation in ovarian cancer.

Scotton and colleagues reported that ovarian tumors were positive for SDF-1 in immunohistochemical staining. Interestingly, benign mucinous tumors were negative and benign serous tumors (which are related to serous cancers) were positive for this chemokine.^29^ Another study demonstrated a high expression of SDF-1 in human ovarian tumor cells that accumulate plasmacytoid dendritic cells in tumor microenvironment.^30^ These dendritic cells induce neoangiogenesis by IL-8 and TNF-α.^31^ Several studies have indicated that SDF-1 is involved in cancer cell migration and metastasis.^32^^,^^33^Consistently, Fushimi demonstrated that the gene transfer of SDF-1α to mice tumors can accumulate dendritic cells and tumor specific cytotoxic T lymphocytes (CTL) in the tumor for the inhibition of tumor growth.^16^ Besides, SDF-1 has other physiological functions such as mediating osteoclast and neural progenitor cells trafficking.^34^^,^^35^ To consider antitumor function of SDF-1, we hypothesized that lower expression of SDF-1α in malignant tissues may have an important role in ovarian tumor growth. However, with regard to the contradictory role of SDF-1 and the higher expression of SDF-1α in benign cysts (in this study), this hypothesis requires more scientific proof. On the other hand, it is speculated that higher expression of SDF-1α in benign cysts may be related to an unknown pathological role in the process of cyst formation, which in the long term depends on the host susceptibility and may contribute to the neoplastic transformation of ovarian epithelial cells. However, the lower expression of IL-4 in ovarian tumor tissues should be interpreted with caution.

Immunosuppressive cytokines such as IL-10, suppress immune response in ovarian tumor microenvironments through inducing several mechanisms such as B7-H4 expression on macrophages and the apoptosis of these cells.^36^ In ovarian cancer, this critical cytokine is not redundant with other suppressive factors and the blockade of the IL-10 signaling network leads to improved survival.^37^ Another study has suggested that IL-10 in combination with CA-125 may be useful in the diagnosis and also the monitoring of chemotherapy results in patients with ovarian carcinoma. However, if more samples had been collected in this study, the differences might have become significant. Higher levels of CA125 and IL-10 in the serum of patients with ovarian cancer compared to patients with ovarian cysts might indicate that the combination of these biomarkers could be used for distinguishing patients with ovarian cancer from those with benign cysts. However, more investigation with higher number of samples is recommended for future studies.

## Conclusion

Overall, lower expression of SDF-1 in malignant tissues may have an important role in ovarian tumor growth. With regard to the contradictory role of SDF-1 in other studies and the higher expression of SDF-1 in benign cysts (in our results), this hypothesis requires more investigation. Additionally, non-significant higher expression of CXCR-4 in ovarian tissue of Iranian patients with malignant cancer in comparison to those with benign cysts might be related to the probable role of this receptor in ovarian cancer promotion. Regarding previous studies in other populations, our result might have become significant using a higher sample size. Significant lower expression of IL-4 in malignant ovarian tissues compared to benign cysts suggests the need for more studies in the future to reveal if this cytokine has an antitumor role in ovarian cancer. Higher levels of CA125 and IL-10 in the serum of patients might indicate that the combination of these biomarkers could be used for distinguishing patients with ovarian cancer from those with benign cysts. 
